# Similar Personality Patterns Are Associated with Empathy in Four Different Countries

**DOI:** 10.3389/fpsyg.2016.00290

**Published:** 2016-03-08

**Authors:** Martin C. Melchers, Mei Li, Brian W. Haas, Martin Reuter, Lena Bischoff, Christian Montag

**Affiliations:** ^1^Department of Psychology, University of BonnBonn, Germany; ^2^Student Counseling Center, Beijing University of Civil Engineering and ArchitectureBeijing, China; ^3^Department of Psychology, University of GeorgiaAthens, GA, USA; ^4^Center for Economics and Neuroscience, University of BonnBonn, Germany; ^5^Institute of Psychology and Education, Ulm UniversityUlm, Germany

**Keywords:** empathy, personality, Big Five, culture comparison, emotion

## Abstract

Empathy is an important human ability associated with successful social interaction. It is currently unclear how to optimally measure individual differences in empathic processing. Although the Big Five model of personality is an effective model to explain individual differences in human experience and behavior, its relation to measures of empathy is currently not well understood. Therefore, the present study was designed to investigate the relationship between the Big Five personality concept and two commonly used measures for empathy [Empathy Quotient (EQ), Interpersonal Reactivity Index (IRI)] in four samples from China, Germany, Spain, and the United States of America. This approach was designed to advance the way the Big Five personality model can be used to measure empathy. We found evidence of medium effect sizes for associations between personality and empathy, with agreeableness and conscientiousness as the most important predictors of affective and cognitive empathy (measured by the respective IRI subscales) as well as for a one-dimensional empathy score (measured by the EQ). Empathy in a fictional context was most closely related to openness to experience while personal distress was first of all related to neuroticism. In terms of culture, we did not observe any distinct pattern concerning cultural differences. These results support the cross-cultural applicability of the EQ and the IRI and indicate structurally similar associations between personality and empathy across cultures.

## Introduction

Empathy represents an important human social construct enabling successful social interactions (e.g., Ford, 1982). In the broadest sense, empathy is defined as the reactions of one individual to the observed experiences of another (Davis, 1983), although this is only one of many definitions discussed in the literature (e.g., Walter, 2012). Despite a large body of research designed to understand empathy, the way empathy is conceptualized is currently not well understood. For example, there currently exist several points of discussions concerned with disentangling (sub)components of empathy (e.g., Batson, 2009), its measurement (e.g., Melchers et al., 2015) or possible causes for individual differences (e.g., Moore, 1990; Knafo et al., 2008). This study was designed to help resolve these issues, in part, by investigating the association between the five-factor model of personality and trait empathy across cultures.

Personality is one effective way to measure individual differences in cognitive thinking patterns or emotional tendencies. The Big Five personality model has developed as one of the most representative models of personality structure (Deary and Matthews, 1993; McCrae and Costa, 2003). Although the Big Five are associated with a variety of processes and concepts related to empathy like attachment styles and romantic relationship outcomes (Shaver and Brennan, 1992), emotional intelligence (Van der Zee et al., 2002), or impulsivity (Whiteside and Lynam, 2001), there are, to our knowledge, only three studies dealing with the relationship of empathy with the Big Five.

Wakabayashi and Kawashima (2015) investigated the relationship between the NEO-FFI (Costa and McCrae, 1992) and empathy as measured by the Empathy Quotient (Baron-Cohen and Wheelwright, 2004; EQ) in a sample of Japanese university students. The authors found nearly no relationship (only 2–3% explained variance in terms of overlap) and associations were only significant in case of prediction via agreeableness or extraversion. The authors interpreted their results in parts by cultural factors. In addition, they argue that the EQ was designed to measure cognitive empathy (i.e., the ability to take the perspective of another person), which (according to the authors) is not closely related to more affective personality traits like, for example, agreeableness. In contrast, Nettle (2007) found strong associations between the Big Five and the EQ. In his study, data were collected by use of an online questionnaire in the USA, and the Big Five were assessed using the International Personality Item Pool’s IPIP five-factor personality scales (Goldberg, 1999). Nettle (2007) observed that agreeableness (*r* = 0.75) and extraversion (*r* = 0.37) were significantly associated with higher EQ values. Nettle raised the question whether agreeableness and empathy may be the same general concept, although one must keep in mind that the reported size of correlation means that still more than 40% (1–0.75^2^) of variance between both questionnaires was not shared. Finally, del Barrio et al. (2004a) investigated a sample of Spanish adolescents. Empathy was assessed with the Spanish version of the Bryant Empathy Index for Children and Adolescents (Bryant, 1982; del Barrio et al., 2004b), and the Big Five were measured with the Spanish version of the Big Five Questionnaire (Caprara et al., 1993), in which some dimensions are named differently compared to those in the classic inventories NEO-FFI or NEO-PI-R. The authors found medium size relations between empathy and the Big Five, especially to friendliness (agreeableness), openness, and conscientiousness. Overall, these results do not provide a complete understanding of the association between personality and empathy that may, in part, be influenced by cultural differences as well as by differences due to inconsistent measurement methods.

An important aspect to consider when comparing samples from different cultures is the cross-cultural applicability of the Big Five. The Big Five have been translated and used in numerous languages under the assumption of cross-cultural applicability (Schmitt et al., 2007). Despite this frequent use, some researchers are skeptical whether it is possible to use the same questionnaire within different cultural contexts (e.g., Poortinga and Van Hemert, 2001). And indeed, there are a number of concerns and problems that need to be kept in mind when using Big Five measures within different cultural contexts: For example, it has been discussed whether the personality trait taxonomy, which is the basis for the Big Five structure, can be applied to different languages, because studies provided evidence for structural inhomogeneities depending on language (e.g., De Raad, 1998) and there are authors who assume language specific idiosyncrasies (e.g., Juni, 1996). On the other hand, studies testing the generalizability of the Big Five factor solution have replicated the five-factor structure quite well for many languages (Church and Lonner, 1998; Jolijn Hendriks et al., 2003). Another problem concerns whether data raised in different cultural contexts possess scalar equivalence (e.g., Byrne and Campbell, 1999) or whether encountered differences may be the result of matching translations, uneven sampling or culture-specific style of responding (Schmitt et al., 2007). Besides, different cultural groups may respond depending on different reference groups, who in turn may influence their response patterns (e.g., Heine et al., 2002). Despite these and many other difficulties (for a more detailed portrayal compare for example Van de Vijver and Leung, 2001; McCrae and Allik, 2002), new research methods and strategies for data analysis allow meaningful cultural comparisons: these safety measures (see also Schmitt et al., 2007) include, for example, specific guidelines for the translation of questionnaires (e.g., Beaton et al., 2000), the use of structural equation models (e.g., Cheung and Rensvold, 2000) or the implementation of new scales or items to improve the comparability of questionnaires (e.g., van de Vijver, 2000).

Initial research on the cross-cultural applicability of the Big Five (e.g., John and Srivastava, 1999) has shown replicability for the Germanic languages and has drawn a more diverse picture for non-Western cultures and languages, especially questioning the universality of the openness dimension. Besides, research on the NEO-PI-R has depicted the cultural sensitivity of agreeableness and extraversion, because some of their facets load on other dimensions depending on culture (McCrae and Allik, 2002). More recent research found configural but no factorial or scalar invariance for the Big Five (Nye et al., 2008) and noticeable differences in item functioning (Church et al., 2011). Besides, some researchers found evidence for additional indigenous factors like positive and negative evaluation (Almagor et al., 1995; Benet and Waller, 1995) or Chinese tradition (Cheung and Leung, 1998). In sum (see also McCrae and Allik, 2002), the cross-cultural validity of the dimensions extraversion, agreeableness and conscientiousness seems clearly established although extraversion and agreeableness are culture-sensitive. For neuroticism, intercultural applicability is also hedged, although in this case results also depend on the methodological approach (etic vs. emic, see also Davidson et al., 1976). The cross-cultural validity of the openness dimension is questionable to an extent which makes some authors even argue for dropping the dimension. But although there are problems and concerns when using the Big Five model in cross-cultural research, the model still is “*the best working hypothesis of an omnipresent trait structure”* (De Raad et al., 1998).

Based on the previous studies concerning the empathy – personality relationship, the current study follows two major aims: first, to address the question whether (and how) the Big Five personality model is associated with empathy. Furthermore, because previous studies have utilized inconsistent measurement approaches across different cultures, we investigated the effect of culture on the association between personality and empathy by using similar measurement tools (set of questionnaires) across samples (China, Germany, Spain, and the United States of America). The design of this approach may help to explain the inconsistent results reported in previous studies, and may also shed light on general differences concerning questionnaire responses across cultures. For the measurement of empathy, we selected two of the most commonly used measures, the Interpersonal Reactivity Index (IRI; Davis, 1983) and the EQ. The IRI measures cognitive (i.e., the ability to put oneself in the shoes of another person) and affective (i.e., the ability to feel with another person) aspects of empathy on separate scales [as well as empathy in a fictional context (fantasy) and distress triggered by social interaction (personal distress)]. The EQ measures all empathy components in one score. We measured the Big Five by using the NEO-FFI.

Based on prior studies demonstrating associations between the Big Five and empathy, we predict to observe positive associations between empathy measures (EQ as well as the cognitive and affective subscale of the IRI) and agreeableness, conscientiousness, openness, and extraversion. In terms of culture, we do not predict major differences across cultures. Due to the heterogeneity of previous mono-cultural results and because of the lack of studies comparing the empathy – personality relationship between cultures, we refrain from any specific predictions concerning culture.

## Materials and Methods

### Participants

We obtained data in China [*N* = 438; *M*_(age)_ = 19.61; *n* = 167 women], Germany (*N* = 304; *n* = 232 women; *M*_(age)_ = 21.17], Spain^1^ (*N* = 62; *n* = 35 women; *M*_(age)_ = 28.02] and the United States of America (*N* = 92; *n* = 56 women; *M*_(age)_ = 19.58] by use of an self-constructed online questionnaire based on the Adobe Go Live software. The questionnaire was designed in such way that participants could not send back incomplete data. All data was collected within a university environment. Participants (mainly undergraduates) were invited to participate via online-advertisement and in classes. Participants received no monetary compensation for their participation but credits (as part of their study regulations). Written informed consent to participate was obtained prior to testing, and the study was approved by the local ethics committee at the Universities of Georgia, USA and Bonn, Germany.

### Measures

In this study, self-reported empathy is measured with the IRI (Davis, 1983) and the EQ (Baron-Cohen and Wheelwright, 2004), because both are among the most common used questionnaires in studies on empathy.

The IRI consists of the four subscales empathic concern, perspective taking, fantasy and personal distress. According to Davis (1983), these exhibit orthogonality, which is why they are not aggregated into a total score. The first two subscales measure affective (empathic concern; EmC) and cognitive (perspective taking; PeT) aspects of empathy. Fantasy (Fan) grasps subjects’ ability to transpose themselves into feelings, thoughts and actions of fictional characters, and personal distress (PeD) has been defined as the “self-oriented” feelings of personal anxiety and unease in tense interpersonal settings (Davis, 1983). Translated versions of the questionnaire were used [Siu and Shek (2005) for the Chinese version, Enzmann (1996) for the German version and Pérez-Albéniz et al. (2003) for the Spanish version]. Final questionnaire versions were inspected by native speakers and retranslated into English as an additional quality check.

The EQ (Baron-Cohen and Wheelwright, 2004, p. 166) has been developed in the context of research on autism as a screening device to measure empathic abilities. It yields a general empathy score, which does not differentiate between facets of empathy (as done in the IRI), because the authors argue that although a cognitive and an affective side of empathy exist, both co-occur in most instances and therefore cannot be easily disentangled. The EQ does, however, exhibit a three-dimensional structure with underlying factors called cognitive empathy, emotional reactivity and social skills (Lawrence et al., 2004; Muncer and Ling, 2006), which can be measured separately. Translated versions of the questionnaire were taken from www.autismresearchcentre.com/arc_tests. Final questionnaire versions were inspected by native speakers and retranslated into English as an additional quality check. For more details concerning the empathy measures, please see also Melchers et al. (2015).

The NEO-FFI questionnaire (Costa and McCrae, 1992) is one of the most common multidimensional inventories assessing the five most important personality domains extraversion, neuroticism, openness to experience, conscientiousness and agreeableness according to the lexical approach to personality. These domains are measured on the level of interval scaling. The NEO-FFI consists of 60 items and has proven good reliability and validity. In case of the Spanish and the USA sample, values for the NEO-FFI were calculated from the longer NEO-PI-R version. Translated versions of the questionnaire were used [McCrae et al. (1996) for the Chinese version, Borkenau and Ostendorf (1993) for the German version and Aluja et al. (2002) for the Spanish version]. Final questionnaire versions were inspected by native speakers and retranslated into English as an additional quality check.

### Statistical Methods

Initially, we checked for associations of empathy to age and gender in all samples by use of correlations and analyses of variance (ANCOVA), respectively. These analyses were conducted, because gender (e.g., Schmitt et al., 2008; Derntl et al., 2010) as well as age (e.g., Lennon and Eisenberg, 1987; Roberts et al., 2006) have been shown to impact on personality and empathy. This is particularly important, because our samples were not equal concerning these variables (compare results section). Hereafter, we performed sensitivity analyses for the samples by use of the G^∗^Power 3.1 software (Faul et al., 2009). We did so because of the varying sample size, which lead to differences in sensitivity between samples. For these analyses, we used t-tests with α = 0.01, power = 0.80 and the respective sample size as input parameters. Next, we investigated the relationship between empathy and personality in the total sample by use of Pearson’s correlations, and by use of stepwise hierarchical regressions to predict EQ values or one of the IRI dimensions, respectively, by use of the Big Five. The latter analyses were performed to identify the most important predictors for empathy and to account for multicollinearity between the five dimensions of the NEO-FFI. Subsequently, we investigated whether there were mean differences in questionnaire responses between samples by use of ANCOVA, controlling for age and including gender as a second factor. Finally, we analyzed and compared the relationship between personality and empathy for each sample in the same manner as we analyzed the data across samples. In case of the correlations, we further analyzed whether there are significant differences in amount of correlation between cultures by use of z statistics. Besides, we once again performed sensitivity analyses by use of the G^∗^Power 3.1 software. For these analyses, we used *z*-tests (Cohen’s q; Rosnow and Rosenthal, 2003) with α = 0.01, power = 0.80 and the respective sample sizes as input parameters. In case of the regression models, we used a stepwise forward selection approach with gender and age as a first step and the Big Five dimensions as a second step. For all analyses, a significance threshold of *p* = 0.01 was defined to account for multiple testing of the five personality dimensions (*p* = 0.05 divided by five tests).

## Results

### Age, Gender, and Sensitivity

Descriptive data concerning gender distribution and mean age are presented in **Table 1**. In both cases, there were highly significant differences between our four samples. In case of age, all *post hoc* comparisons except the China/USA comparison delivered significant results. In case of gender, especially the distribution in the Chinese sample (more male than female participants) differed from the other nations.

**Table 1:** Means, (standard deviations), [Cronbach’s Alpha] and ANCOVA for the questionnaire responses of the four samples after inserting age as a covariate and including gender as a second independent variable.

	Whole sample	China	Germany	Spain	USA	Country comparison
Empathy Quotient	42.14 (11.08) [0.86]	39.32^1^ (10.41) [0.84]	44.44^1,2^ (10.47) [0.88]	43.21 (11.09) [0.85]	40.39^2^ (10.69) [0.86]	*F*_(3,887)_ = 11.437, *p* < 0.001
IRI fantasy	17.25 (5.63) [0.55]	16.71 (4.79) [0.84]	16.76 (6.70) [0.80]	18.70 (5.74) [0.80]	17.93 (5.58) [0.86]	n.s.
IRI perspective taking	17.79 (4.46) [0.72]	17.73 (4.72) [0.70]	17.49 (4.05) [0.76]	18.78 (4.16) [0.70]	17.46 (4.48) [0.81]	n.s.
IRI empathic concern	19.58 (4.13) [0.66]	19.60 (4.20) [0.71]	18.64^1^ (3.68) [0.72]	20.79^1^ (4.07) [0.68]	19.11 (4.14) [0.78]	*F*_(3,887)_ = 5.302, *p* < 0.001
IRI personal distress	12.09 (4.62) [0.67]	11.73 (4.91) [0.68]	12.07 (4.06) [0.71]	12.09 (4.25) [0.75]	12.74 (4.59) [0.80]	n.s.
NEO extraversion	3.38 (0.54) [0.74]	3.29^1,2^ (0.48) [0.70]	3.47^1^ (0.59) [0.78]	3.52^2^ (0.57) [0.81]	3.40 (0.54) [0.83]	*F*_(3,887)_ = 6.314, *p* < 0.001
NEO neuroticism	2.88 (0.69) [0.83]	2.84^1^ (0.68) [0.86]	2.76^2^ (0.67) [0.87]	2.96 (0.67) [0.84]	3.18^1,2^ (0.68) [0.89]	*F*_(3,887)_ = 8.997, *p* < 0.001
NEO openness	3.48 (0.50) [0.69]	3.37^1,2^ (0.47) [0.61]	3.70^1,3^ (0.51) [0.72]	3.65^2,4^ (0.45) [0.66]	3.30^3,4^ (0.48) [0.62]	*F*_(3,887)_ = 26.195, *p* < 0.001
NEO conscientiousness	3.55 (0.58) [0.84]	3.46^1,2^ (0.54) [0.80]	3.65^1,3,4^ (0.58) [0.86]	3.22^2,3,5^ (0.68) [0.86]	3.55^4,5^ (0.57) [0.87]	*F*_(3,887)_ = 10.613, *p* < 0.001
NEO agreeableness	3.66 (0.48) [0.69]	3.62^1^ (0.47) [0.65]	3.72^2^ (0.46) [0.71]	3.73^3^ (0.51) [0.72]	3.39^1,2,3^ (0.47) [0.71]	*F*_(3,887)_ = 11.257, *p* < 0.001
Age	20.72 (4.58)	19.58^1,2^ (1.82)	21.64^1,3,4^ (4.27)	27.72^2,3,5^ (11.07)	19.53^4,5^ (1.81)	*F*_(2,883)_ = 79.611, *p* < 0.001
Gender (women/men)	490/408	167/271	232/72	35/28	56/36	Chi^2^ = 107.353, *p* < 0.001

*Index numbers indicate significant post hoc tests, numbers with the same index number (per line) differ significantly from each other (corrected with Bonferroni).*

Analyses of the relations between age and empathy/persona lity revealed the following results: in the Chinese sample, age was negatively related to Fan (*r* = -0.151, *p* = 0.002), neuroticism (*r* = -0.109, *p* = 0.023), openness (*r* = -0.101, *p* = 0.035) and agreeableness (*r* = -0.114, *p* = 0.017). In the German sample, negative correlations of age were found with Fan (*r* = -0.143, *p* = 0.012) and PeD (*r* = -0.139, *p* = 0.018). In the Spanish sample, age was negatively associated with Fan (*r* = -0.333, *p* = 0.008), openness (*r* = -0.414, *p* = 0.001) and positively to conscientiousness (*r* = 0.313, *p* = 0.013). Finally, in the sample from the USA there was only a negative correlation between age and neuroticism (*r* = -0.268, *p* = 0.010).

Concerning gender, there was only one significant difference in the Chinese sample: Chinese women report more stress in social interaction than men [*F*_(1,436)_ = 16.282, *p* < 0.001]. In contrast, we found many differences depending on gender in the German sample: women depicted higher values in all empathy dimensions except perspective taking [Fan: *F*_(1,302)_ = 13.894, *p* < 0.001; EmC: *F*_(1,302)_ = 42.771, *p* < 0.001; PeD: *F*_(1,302)_ = 21.439, *p* < 0.001; EQ: *F*_(1,302)_ = 28.711, *p* < 0.001] as well as in neuroticism [*F*_(1,302)_ = 8.609, *p* = 0.004], agreeableness [*F*_(1,302)_ = 24.094, *p* < 0.001] and conscientiousness [*F*_(1,302)_ = 12.044, *p* = 0.001]. In the Spanish sample, significant differences were observed for the EQ [*F*_(1,60)_ = 10.540, *p* = 0.002], openness [*F*_(1,60)_ = 9.227, *p* = 0.004] and agreeableness [*F*_(1,60)_ = 4.609, *p* = 0.036]. Women scored higher in empathy and agreeableness while men scored higher in openness. Finally, in the sample from the USA we observed no significant gender differences.

Analyses concerning our samples’ sensitivity revealed the following results: Given the respective samples sizes, an α = 0.01 and an expected power of 0.80, we could detect correlation coefficients down to *r* = 0.161 for the Chinese sample (*t* = 2.587, *df* = 436), r = 0.193 for the German sample (*t* = 2.592, *df* = 303), *r* = 0.408 for the Spanish sample (*t* = 2.660, *df* = 60) and *r* = 0.341 for the US-American sample (*t* = 2.632, *df* = 90).

### Association Between Personality and Empathy in the Total Sample

Mean questionnaire responses and standard deviations across all four subsamples are presented in **Table 1.** Overall, these are comparable to the responses found in other studies administering the IRI, the EQ or the NEO.

**Table 2** depicts the partial correlations between empathy and personality (corrected for age) in the total sample. Here, agreeableness is the personality dimension demonstrating the highest correlation with the EQ as well as with the affective and the cognitive empathy subscale from the IRI. For fantasy, openness is the personality dimension with the highest correlation, for personal distress it is neuroticism. Analyses of gender differences in these correlations depicted no significant differences.

**Table 2:** Partial correlations (corrected for age) between measures for Empathy and the Big Five across all four samples.

	Extraversion		Neuroticism		Openness		Conscientiousness		Agreeableness	
	*r*	*p*	*r*	*p*	*r*	*p*	*r*	*p*	*r*	*p*
Empathy Quotient	0.306	<0.001	-0.149	<0.001	0.249	<0.001	0.408	<0.001	0.467	<0.001
IRI fantasy	0.073	0.030	0.189	<0.001	0.240	<0.001	–0.015	0.663	0.023	0.894
IRI perspective taking	0.117	<0.001	–0.145	<0.001	0.202	<0.001	0.243	<0.001	0.334	<0.001
IRI empathic concern	0.152	<0.001	0.067	0.045	0.090	0.007	0.141	<0.001	0.348	<0.001
IRI personal distress	–0.166	<0.001	0.553	<0.001	–0.061	0.068	–0.199	<0.001	–0.042	0.207

*Coefficients were also calculated for the female and the male subsample (compare results). There were no significant gender differences in any of the correlations. A significance threshold of p = 0.01 was defined to account for multiple testing of the five personality dimensions.*

**Table 3** shows the hierarchical regression models predicting either the EQ or one of the IRI subscales by gender and age (first step) and personality (second step) in a forward selection approach model. Results once again show that agreeableness (and in case of the EQ to a lesser extend conscientiousness) is the most important personality dimension to predict the classical empathy dimensions. The relationships between openness and fantasy as well as between neuroticism and personal distress are still highly significant and keep about the same effect size although the regression model controls for potential effects of multicollinearity. Overall, the EQ or personal distress, respectively, share about double the amount of variance with personality than perspective taking or empathic concern. Fantasy, in comparison, is only predicted poorly.

**Table 3:** Results of the hierarchical regression models predicting the EQ or a subscale of the IRI, respectively, with the Big Five dimensions across all samples.

	Gender and age	Extraversion	Neuroticism	Openness	Conscientious-ness	Agreeableness	Complete Big Five
Empathy Quotient	*p* < 0.001, 5.4%	*p* < 0.001, 1.2%	*p* = 0.010, 0.4%	*p* < 0.001, 3.8%	*p* < 0.001, 7.5%	*p* < 0.001, 18.5%	*p* < 0.001, 31.4%
IRI fantasy	*p* < 0.001, 2.7%	*p* < 0.001, 1.7%	*p* < 0.001, 3.2%	*p* < 0.001, 5.3%	n.s.	n.s.	*p* < 0.001, 10.2%
IRI perspective taking	n.s.	n.s.	n.s.	*p* < 0.001, 3.4%	*p* < 0.001, 1.7%	*p* < 0.001, 11.2%	*p* < 0.001, 16.3%
IRI empathic concern	n.s.	*p* < 0.001, 1.4%	*p* < 0.001, 3.3%	n.s.	n.s.	*p* < 0.001, 12.2%	*p* < 0.001, 16.9%
IRI personal distress	*p* < 0.001, 5.6%	n.s.	*p* < 0.001, 28.9%	*p* = 0.001, 0.9%	*p* = 0.009, 0.4%	*p* < 0.001, 0.8%	*p* < 0.001, 30.8%

*Predictors were entered by use of a stepwise forward selection approach with gender and age as first step followed by the Big Five in a second step. The table presents significance values as well as explanation of variance of each predictor and significance values and explanation of variance for the entire model (all Big Five dimensions, the last column results from adding the Big Five dimensions – e.g., 1.2 + 0.4 + 3.8 + 7.5 + 18.5 = 31.4%).*

*Note that the first column includes the sum of variance explained by gender and age. Individual values may also present only one of the variables if the other didn’t significantly predict values in personality.*

### Comparison of Self-Report Measures Between Samples

Mean questionnaire responses and standard deviations of the four samples are also presented in **Table 1.** With the exception of perspective taking, fantasy and personal distress, there were significant differences between countries for all administered questionnaires (corrected for age and with gender as a second factor). Most significant differences were found between the USA and Germany (5), followed by China and Germany (4) and Spain and China/the USA (3 each).

### Association Between Personality and Empathy Between Cultures

**Table 4** depicts the correlations between the Big Five and the empathy measures for all four samples.

**Table 4:** Partial correlations between measures for Empathy and the Big Five for the Chinese (C), German (D), Spanish (E) and USA sample corrected for age.

		Extraversion		Neuroticism		Openness		Conscientiousness		Agreeableness	
		*r*	*p*	*r*	*p*	*r*	*p*	*r*	*p*	*r*	*p*
Empathy Quotient	C	0.3311	<0.001	–0.197	<0.001	0.262	<0.001	0.405	<0.001	0.385	<0.001
	D	0.1931	0.001	–0.123	0.031	0.137	0.017	0.369	<0.001	0.489	<0.001
	E	0.185	0.153	–0.107	0.411	0.017	0.899	0.209	0.105	0.468	<0.001
	USA	0.359	<0.001	–0.096	0.366	0.178	0.091	0.445	<0.001	0.532	<0.001
IRI fantasy	C	0.065	0.175	0.262	<0.001	0.193^1,2^	<0.001	0.036	0.452	0.006	0.896
	D	0.074	0.199	0.075	0.193	0.211	<0.001	–0.070	0.220	–0.047	0.418
	E	0.120	0.355	0.117	0.370	0.453^1^	<0.001	–0.189	0.145	0.206	0.112
	USA	–0.076	0.474	0.308	0.003	0.408^2^	<0.001	0.099	0.349	0.163	0.123
IRI perspective taking	C	0.215	<0.001	–0.164	<0.001	0.258	<0.001	0.327^1^	<0.001	0.353	<0.001
	D	0.057	0.325	–0.164	0.004	0.143	0.013	0.177^1^	0.002	0.377	<0.001
	E	0.172	0.184	–0.091	0.483	0.166	0.203	0.096	0.463	0.259	0.044
	USA	0.111	0.295	–0.074	0.484	0.237	0.023	0.327	0.002	0.263	0.012
IRI empathic concern	C	0.243	<0.001	0.089	0.064	0.049	0.303	0.149	0.002	0.291	<0.001
	D	0.077	0.179	0.054	0.350	0.137	0.017	0.189	<0.001	0.468	<0.001
	E	0.020	0.878	0.117	0.371	0.164	0.206	0.099	0.448	0.401	0.001
	USA	0.120	0.256	–0.006	0.952	0.134	0.206	0.198	0.059	0.352	<0.001
IRI personal distress	C	–0.177	<0.001	0.594	<0.001	–0.144	0.003	–0.335	<0.001	–0.140	0.003
	D	–0.219	<0.001	0.536	<0.001	–0.014	0.811	–0.098	0.087	0.022	0.701
	E	–0.218	–0.092	0.489	<0.001	0.092	0.479	–0.059	0.652	0.168	0.196
	USA	–0.191	0.070	0.488	<0.001	–0.044	0.680	–0.258	0.013	0.004	0.973

Results have been corrected for age. To be able to detect gender differences, coefficients were calculated separately for female and male participants. The comparison of the relationship of personality to empathy between samples revealed only three significant differences: for the EQ and extraversion (China/Germany), for perspective taking and conscientiousness (China/Germany) and for fantasy and openness (China/Spain/USA). It should be noted, however, that the power to detect differences between samples varies depending on the respective sample combination due to the differences in sample size (compare methods), as can be derived from the results of our additional sensitivity analysis. Given an α = 0.01, power = 0.80 and the respective sample sizes, we could detect the following differences between correlation coefficients: China/Germany: *q* = 0.256; China/Spain: *q* = 0.474; China/USA: *q* = 0.398; Germany/Spain: *q* = 0.486; Germany/USA: *q* = 0.412; Spain/USA: *q* = 0.574.

**Table 5** shows the results of the hierarchical regressions, which account for multicollinearity between the Big Five dimensions. Overall, the Big Five predicted 14–46% of the responses given to the EQ dependent on country subsample. For the IRI, results are more mixed. With respect to the affective and cognitive component of empathy and the fantasy scale, the Big Five explain between 5 and 22% of variance, for personal distress much more (24–36%). There is substantial variation between cultures in the predictive power of the models trying to predict the same empathy dimension. However, if comparing this variation between the empathy dimensions, there is no uniform pattern (i.e., there is no culture where in general personality explains more or less variation in empathy than in another culture). Here, we once again must keep in mind the differences in sample sizes and its impact on power. From a content perspective, the “classical” empathy dimensions (EQ, IRI perspective taking, empathic concern) seem primarily related to agreeableness (and in addition conscientiousness in case of the EQ), while fantasy is mainly associated to openness and personal distress mainly to neuroticism. Despite differences in the variance explained, the order of predictors is remarkably consistent across samples.

**Table 5:** Results of the hierarchical regression models predicting the EQ or a subscale of the IRI, respectively, with the Big Five dimensions for the Chinese (C), German (D), Spanish (E), and USA sample.

		Gender and age	Extraversion	Neuroticism	Openness	Conscientious-ness	Agreeableness	Complete Big Five
Empathy	C	n.s.	*p* < 0.001, 1.9%	n.s.	*p* < 0.001, 3.4%	*p* < 0.001, 16.1%	*p* < 0.001, 7.7%	*p* < 0.001, 29.1%
Quotient	D	*p* = 0.001, 8.7%	n.s.	n.s.	*p* < 0.001, 3.8%	*p* < 0.001, 4.6%	*p* < 0.001, 18.4%	*p* < 0.001, 26.8%
	E	n.s.	n.s.	n.s.	n.s.	n.s.	*p* < 0.001, 14.3%	*p* < 0.001, 14.3%
	USA	n.s.	*p* = 0.003, 3.7%	n.s.	*p* = 0.008, 4.6%	*p* = 0.010, 9.2%	*p* < 0.001, 28.1%	*p* < 0.001, 45.6%
IRI fantasy	C	n.s.	*p* < 0.001, 3.2%	*p* < 0.001, 6.7%	*p* < 0.001, 4.1%	n.s.	n.s.	*p* < 0.001, 14.0%
	D	*p* < 0.001, 4.4%	n.s.	n.s.	*p* < 0.001, 4.5%	n.s.	n.s.	*p* < 0.001, 4.5%
	E	*p* < 0.001, 11.1%	n.s.	n.s.	*p* < 0.001, 18.3%	n.s.	n.s.	*p* < 0.001, 18.3%
	USA	n.s.	n.s.	n.s.	*p* = 0.001, 16.5%	n.s.	n.s.	*p* < 0.001, 16.5%
IRI	C	n.s.	n.s.	n.s.	*p* < 0.001, 6.0%	*p* < 0.001, 3.1%	*p* < 0.001, 12.5%	*p* < 0.001, 21.6%
perspective	D	n.s.	n.s.	n.s.	*p* = 0.001, 2.9 %	n.s.	*p* < 0.001, 14.3%	*p* < 0.001, 17.2%
taking	E	n.s.	n.s.	n.s.	n.s.	n.s.	n.s.	n.s.
	USA	n.s.	n.s.	n.s.	n.s.	*p* = 0.002, 10.3%	n.s.	*p* = 0.001, 10.3%
IRI empathic	C	n.s.	*p* < 0.001, 4.4%	*p* < 0.001, 4.4%	n.s.	*p* = 0.004, 1.5%	*p* < 0.001, 8.2%	*p* < 0.001, 18.5%
concern	D	*p* < 0.001, 12.4%	n.s.	n.s.	*p* < 0.001, 3.3%	n.s.	*p* < 0.001, 15.8%	*p* < 0.001, 19.1%
	E	n.s.	n.s.	n.s.	n.s.	n.s.	*p* = 0.001, 15.9%	*p* = 0.001, 15.9%
	USA	n.s.	n.s.	n.s.	n.s.	n.s.	*p* = 0.001, 11.8%	*p* = 0.001, 11.8%
IRI personal	C	*p* < 0.001, 3.6%	n.s.	*p* < 0.001, 34.8%	*p* = 0.005, 1.6%	n.s.	n.s.	*p* < 0.001, 36.4%
distress	D	n.s.	n.s.	*p* < 0.001, 25.2%	n.s.	n.s.	n.s.	*p* < 0.001, 25.2%
	E	n.s.	n.s.	*p* < 0.001, 24.2%	n.s.	n.s.	n.s.	*p* < 0.001, 24.2%
	USA	n.s.	n.s.	*p* < 0.001, 26.5%	n.s.	n.s.	n.s.	*p* < 0.001, 26.5%

*Predictors were entered by use of a stepwise forward selection approach with gender and age as first step followed by the Big Five in a second step. The table presents significance values as well as explained variance for each predictor and for the entire model (all Big Five dimensions).*

*Note that the first column includes the sum of variance explained by gender and age. Individual values may also present only one of the variables if the other didn’t significantly predict values in personality.*

## Discussion

In this study, we investigated the relationship between the Big Five model of personality and several commonly used measures of empathic processing across samples from four different cultures. Based on previous studies, we hypothesized positive correlations between central measures of empathy and agreeableness, conscientiousness, openness, and extraversion. For the culture dependent analyses, we expected no major differences between cultures.

The observed associations between empathy and personality for the total sample match our expectations. In case of the EQ, but also in case of IRI perspective taking and empathic concern, agreeableness turned out to be the by far most important predictor, explaining between 11 and 18% of variance in empathy responding. For the EQ, conscientiousness was also important (8% explanation of variance) while in case of the two IRI subscales no other personality dimension explained additional substantial amounts of variance. Therefore, our results suggest medium size correlations/associations between empathy and agreeableness, indicating a big importance of personality opposed to Wakabayashi and Kawashima (2015) but not a complete overlay of both constructs opposed to Nettle (2007). Besides, we didn’t observe differences in the prediction of cognitive versus affective empathy, indicating that both associate with the same personality dimensions.

It makes perfectly sense that we found agreeableness to be the best predictor for empathy because it is primarily a dimension of interpersonal behavior and represents the quality of social interaction (Costa et al., 2001). Furthermore, agreeableness can predict prosocial as well as aggressive behavior (Graziano and Eisenberg, 1997). Graziano et al. (2007) even offered a mechanism explaining the association. According to them, humans low in agreeableness do not report less empathy because they lack empathic affect or prosocial motivation, but because they lack skills in shifting the focus of these reactions to others.

From a cross-cultural perspective, research has shown differences in agreeableness between cultures, for example, East Asian cultures have been shown to score the lowest in agreeableness (compare below for a more detailed discussion) and Southern Europeans have depicted higher scores in agreeableness than Eastern and Western Europeans (Schmitt et al., 2007). To our knowledge, there is no comparable detailed study that deals with cultural differences in responding to empathy questionnaires. Therefore, future research with a big number of cultures/countries will show whether the predictive value of agreeableness for empathy on an individual level is also reflected in differences in empathy scores (i.e., lower scores in empathy in East Asian samples, too). In our own data, such a difference is already hinted in the mean score comparison. Here, both significant differences in empathy involve lower scores in China compared to the other samples.

Next to agreeableness, conscientiousness is the second personality dimension with a bigger predictive value for empathy. Here, the association may be explained by the negative relation of conscientiousness to psychoticism (McCrae and Costa, 1985), which is defined by a lack of empathy (Richendoller and Weaver, 1994). This relationship between psychoticism and empathy in turn can be explained by the lack of concern for others as well as egocentricity, which are both among the significant features of psychoticism (Richendoller and Weaver, 1994).

Concerning the other subscales of the IRI, we found neuroticism to be important to explain personal distress and openness to be important to predict fantasy. These results fit in with the IRI’s theoretical background: For both subscales, it has been a matter of discussion whether they are facets of empathy or not (fantasy lacks the personal social interaction, personal distress has been defined as a distinct affective state apart from empathy or as a reaction to a specific empathic makeup, see also Melchers et al., 2015). Therefore, it is plausible that these subscales’ relation to the Big Five differs from the other subscales. For fantasy, a relation to openness is the most plausible, because feeling empathy in a fictional context presupposes openness to the respective fictional world of a book, a theater play etc. Furthermore, openness includes creativity, which also plays an important role in generation and sensitivity to fictional environments. For personal distress, neuroticism fits perfectly as a candidate for an association, because personal distress measures the attitude toward and feelings evoked by negative social interactions. These feelings, in turn, should be strongly influenced by the fundamental attitude to social interactions, which again is strongly related to neuroticism.

Results concerning differences in empathy responses and personality depending on country/culture do not offer a clear cultural trend. Therefore, we refrain from an extensive discussion of these differences, also because they overall suggest cultural similarity as most of the observed differences are small. But what should still be considered is the following: three out of four subscales of the IRI questionnaire show no cultural dependence and even in case of empathic concern the differences are small. Therefore, we expect that the questionnaire can measure cognitive and affective empathy regardless of the culture under investigation.

Analyses concerning the association between the empathy measures and the Big Five indicate homogenous associations across cultures. Overall, only three correlations with significant differences between cultures were found. These data suggests no cultural dependence of the association between empathy and personality. A more nuanced picture emerges when taking results of the regressions, which consider multicollinearity, into account. Here, we see differences in the predictive power of the Big Five for empathy. However, once again there are no clear associations with a specific culture, rather more independent patterns for each subscale are detectable.

Overall, the following picture emerges: We found no big differences between the investigated samples, neither for mean responses (which need to be interpreted very carefully) nor for the relationship between empathy and the Big Five. The differences we found do not follow a uniform pattern related to a putative cultural continuum. Agreeableness and conscientiousness are the most important personality factors to explain empathy responding, openness predicts fantasy and neuroticism personal distress. We found that the Big Five can explain up to 46% of variance in empathy questionnaire responses.

Our results may have important implications for our understanding of empathy as well as for intercultural interaction. Out of an evolutionary perspective, empathy originally developed due to its proximal fitness advantages because it allows non-conflicting or conflict reduced cooperation of individuals in groups by synchronizing or explaining affective and cognitive states, which, for example, brings significant advantages when hunting or defending the own group, but also reduces intra-group conflicts (de Waal, 2008). This development is most likely associated with the increased investment in the own offspring and the associated need for synchronization of affective but also cognitive states between parents and offspring (MacLean, 1985). The need to develop such skills is also reflected in their both ontogenetically and phylogenetically gradual emergence (de Waal, 2008; Walter, 2012). An interesting question is how the later developed cultural differences may have changed the function or the usefulness of empathy for successful social interaction. Here, one should cast a glance to the classic dimensions that have been proposed for the classification of cultures in the literature. Through a large number of cross-cultural studies, Hofstede (1979, 1980, 1982, 1983) was able to identify a total of four dimensions [Power Distance, Uncertainty Avoidance, Individualism vs. Collectivism (InCo) and Masculinity vs. Femininity (MaFe)], according to which cultures can differ from each other. Power Distance has been defined as “the extent to which the less powerful of institutions and organizations accept that power is distributed unequally” (Hofstede and Bond, 1984). Here, one could expect a negative relationship between empathy and high power distance, because the more social interactions or their sequences, respectively, depend on power status, the less empathy is needed to synchronize. Function wise, empathy could be an ability, which helps especially individuals low in power to adapt to the demands of the higher power people. Uncertainty Avoidance is “the extent to which people feel threatened by ambiguous situations, and have created believes and institutions that try to avoid these” (Hofstede and Bond, 1984). In case of this dimension, once could expect a positive relation to empathy, because empathy informs a person of the goals and emotions of a respective interaction partner, thereby reducing ambiguity and creating positive interaction conditions. In other words, empathy (or empathic abilities) should be the more important for a culture, the more uncertainty avoidant the culture is. InCo have been defined as “a situation in which people are supposed to look after themselves and their immediate family only” versus “a situation in which people belong to in-groups or collectivities which are supposed to look after them in exchange for loyalty” (Hofstede and Bond, 1984). Here, expectations could go in both directions: on the one hand, one could expect higher empathy in collectivistic cultures because empathy could serve the function to adapt the individual to their in-group, which is much more important for a collectivistic culture. On the other hand, in individualistic culture there should in general be more variance in behavior compared to a more norm-driven, collectivistic culture, which makes a certain amount of adaption necessary to allow for successful social interaction. Empirical research has given more support to the former hypothesis (e.g., Realo and Luik, 2002; Duan et al., 2008). Studies demonstrating lower self-reported empathy in collectivistic than in individualist cultures (which technically support the latter hypothesis) on the other hand are not easy to interpret, because these mean differences might also be driven by differences in response patterns (compare beyond). Finally, MaFe have been described as “a situation in which the dominant values in society are success, money, and things” versus “a situation in which the dominant values in a society are caring for others and the quality of life” (Hofstede and Bond, 1984). Here, the expected association is rather clear, as the definition of femininity contains a facet of empathy (caring for others). Therefore, one would expect positive correlations of empathy to femininity and negative associations to masculinity. On the other hand, feminine cultures could report more empathy because it is an important value, although there exist no differences in empathic abilities compared to more masculine cultures. A similar difference between self-report and behavior/ability has been reported for general gender differences in empathy (Derntl et al., 2010).

Knowledge on differences depending on cultural properties may be very helpful for our understanding of empathy as well as for practical applications. For the former, the illustration above shows that definitions and functions of empathy may vary depending on the cultural context. This may have, among others, implications for the choice of empathy measures or the interpretation of their results, for example the above-mentioned differences depending on gender. For the latter, knowledge on culture specific empathy is important for example for the design of negotiations (e.g., Buttery and Leung, 1998), the design of customer – service provider relationships (e.g., Dash et al., 2009) or training psychotherapists in cross-cultural empathy (e.g., Dyche and Zayas, 2001).

When interpreting our results, we have to keep in mind that there are some limitations to our findings. One of them concerns the sample sizes for the different samples, which differ between 438 and 62 participants. This difference emerged due to different willingness to participate in the countries under investigation. A consequence is varying power for detecting associations between personality and empathy. This can also be derived from the sensitivity analyses: while we could detect correlations slightly beyond 0.2 in the Chinese and German sample, the US-American sample requires a minimum correlation of 0.34 and the Spanish sample even more (0.4). Especially in case of these latter two we therefore might have not detected the impact of the less strongly associated personality variables. For example, most of the small associations with extraversion were found in the Chinese sample, which is the biggest sample. On the other hand, one could argue that this shortcoming is not a big problem for our study, because these small associations technically don’t play an important role in predicting empathy by personality because they are so small. For example, the biggest correlation between extraversion and the IRI (empathic concern, in the Chinese sample) explains less than 6% variance. After correction for gender, age, and multicollinearity, this effect is even smaller (4.4%).

A second, related argument concerns the gender distribution in the samples. Whilst the Chinese sample contains more male than female participants, the other samples comprise more females than males. For the analyses of mean questionnaire responses, we therefore controlled for age and added gender as a second between-subject factor in addition to culture. We don’t think that the differences distorted results concerning the relationship between personality and empathy, because we found no significant gender related differences in the correlations of empathy and the Big Five (compare results). Besides, our data have been collected in a university setting, which leads to restrictions in age range and education degrees.

Another aspect, which should be reminded in cross-cultural research is the question of comparability of questionnaire responses. Do we really measure the same agreeableness in China and in Germany? Does the IRI measure the same empathy traits in Spain as in the USA? As in our own data, previous research has shown mean differences in questionnaire responses between cultures (e.g., Allik and McCrae, 2004; Schmitt et al., 2007). These studies even supposed patterns in participants’ responses, which could be related to the respective geographic region, thereby creating a kind of map of personality. The interpretation of such differences is difficult: do they represent trait differences, are they caused by differences in response style or social desirability or do they mirror different evaluation of the trait under investigation? And what is the origin of such differences, do they have a cultural or rather a genetic basis or both? For example, Schmitt et al. (2007) found that East Asian participants score lower on all Big Five dimensions except neuroticism (here, they score higher than all other participants) compared to the other groups of participants. The uniformity of this deviation might indicate a response pattern rather than a difference in personality. Allik and McCrae (2004) found smaller standard deviations in Asian and Black African cultures compared to European cultures. This again might be indicative of differences in response patterns, which may impact on the test score. From a content perspective, the Big Five dimensions might also serve different functions in different cultures. For example, the openness factor has not consistently been replicated in China (Cheung et al., 2001), which has been labeled as a collectivistic country (Hofstede, 2001). Openness has been shown to be differentially valued across cultures, and one reason for that might be differences in sociability between collectivistic and individualistic countries (Triandis, 1995; Ward et al., 2004). Another, quite simple, argument could be that openness serves the less a function the more uniform according to given norms an individual is supposed to behave in its culture. A second example for a functional difference is the factor agreeableness. Here, relations have been shown to the idiocentrism (big differences between self and others) versus allocentrism (big differences between own group and other groups) concept (Triandis, 1989) and allocentric individuals/cultures tend to be more agreeable than idiocentric individuals/cultures (Realo et al., 1997). Once again, this might be explained by differences in the function of agreeableness. For example, agreeableness should be more important in an allocentric context to sustain the homogeneity and harmony of a group than in an idiocentric context.

As with personality, cultural differences in questionnaire responses can also be interpreted in different ways in case of empathy. Although there are not many studies in this field, there have already been shown some differences. For example, Trommsdorff (1995) depicted more personal distress and less empathic concern in Japanese (East Asian) children compared to those from Germany (Western culture). Cassels et al. (2010) compared empathy (measured by the IRI) in university undergraduates with an Asian background to those with a Caucasian or a bicultural background. They found greater personal distress and less empathic concern in East Asian participants compared to their Caucasian counterparts and scores of the bicultural participants fell in between. Again, these differences might be the result of different functions of empathy facets depending on culture. One could speculate, for example, whether personal distress evoked by negative social interaction serves a more important function in sustaining harmony and social peace in Asians than in Westerns/Caucasians, while empathic concern in turn serves as the more important function for the same goals in Westerns/Caucasians compared to Asians.

For future studies, it would be useful to collect samples from other countries which include an even more representative assortment of participants and which represent the four descriptive dimensions of cultural differences by Hofstede. Furthermore, it would make sense to consider other measures for empathy and their interaction with the cultures under investigation, as in our case results seem in part to depend on the utilized measure.

## Author Contributions

All authors contributed to preparation and planing of the study. All authors were involved in data collection and interpretation of results. Data analyses and writing of the manuscript was handled by MM. All authors corrected and approved the final manuscript.

## Conflict of Interest Statement

The authors declare that the research was conducted in the absence of any commercial or financial relationships that could be construed as a potential conflict of interest.

## Footnotes

1 Indeed, the Spanish sample differs from the other samples. This is due to a rather low willingness to participate (this is also the reason for the low number of participants compared to the other study sites). At the beginning, we planned only to recruit undergraduates. In case of the Spanish sample we also recruited older students. We tried to account for this difference by correcting all data for age.

## References

[B1] Allik J. McCrae R. R. (2004). Toward a geography of personality traits patterns of profiles across 36 cultures. *J. Cross Cult. Psychol.* 35 13–28. 10.1177/0022022103260382

[B2] Almagor M. Tellegen A. Waller N. G. (1995). The Big Seven model: a cross-cultural replication and further exploration of the basic dimensions of natural language trait descriptors. *J. Pers. Soc. Psychol.* 69 300–307. 10.1037/0022-3514.69.2.300

[B3] Aluja A. García Ó. García L. F. (2002). A comparative study of Zuckerman’s three structural models for personality through the NEO-PI-R, ZKPQ-III-R, EPQ-RS and Goldberg’s 50-bipolar adjectives. *Pers. Individ. Differ.* 33 713–725. 10.1016/S0191-8869(01)00186-6

[B4] Baron-Cohen S. Wheelwright S. (2004). The empathy quotient: an investigation of adults with Asperger syndrome or high functioning autism, and normal sex differences. *J. Autism Dev. Disord.* 34 163–175. 10.1023/B:JADD.0000022607.19833.00 15162935

[B5] Batson C. D. (2009). “These things called empathy: eight related but distinct phenomena,” in *The Social Neuroscience of Empathy*, eds Decety J. Ickes W. (Cambridge, MA: MIT Press), 3–15.

[B6] Beaton D. E. Bombardier C. Guillemin F. Ferraz M. B. (2000). Guidelines for the process of cross-cultural adaptation of self-report measures. *Spine* 25 3186–3191. 10.1097/00007632-200012150-00014 11124735

[B7] Benet V. Waller N. G. (1995). The Big Seven factor model of personality description: evidence for its cross-cultural generality in a Spanish sample. *J. Pers. Soc. Psychol.* 69 701–718. 10.1037/0022-3514.69.6.701

[B8] Borkenau P. Ostendorf F. (1993). *NEO-Fünf-Faktoren Inventar (NEO-FFI) [NEO Five-Factor Inventory].* Göttingen: Psychologie.

[B9] Buttery E. A. Leung T. K. (1998). The difference between Chinese and Western negotiations. *Eur. J. Mark.* 32 374–389. 10.1108/03090569810204652

[B10] Bryant B. K. (1982). An index of empathy for children and adolescents. *Child Dev.* 53 413–425. 10.2307/1128984

[B11] Byrne B. M. Campbell T. L. (1999). Cross-cultural comparisons and the presumption of equivalent mea- surement and theoretical structure: a look beneath the surface. *J. Cross Cult. Psychol.* 30 555–574. 10.1177/0022022199030005001

[B12] Caprara G. V. Barbaranelli C. Borgogni L. Perugini M. (1993). The “Big Five Questionnaire”: a new questionnaire to assess the Five Factor Model. *Pers. Individ. Differ.* 15 281–288. 10.1016/0191-8869(93)90218-R

[B13] Cassels T. G. Chan S. Chung W. Birch S. A. J. (2010). The role of culture in affective empathy: cultural and bicultural differences. *J. Cogn. Cult.* 10 309–326. 10.1163/156853710X531203

[B14] Cheung F. M. Leung K. (1998). Indigenous personality measures Chinese examples. *J. Cross Cult. Psychol.* 29 233–248. 10.1037/a0022389

[B15] Cheung F. M. Leung K. Zhang J. X. Sun H. F. Gan Y. Q. Song W. Z. (2001). Indigenous Chinese personality constructs: is the five-factor model complete? *J. Cross Cult. Psychol.* 32 407–433. 10.1177/0022022101032004003

[B16] Cheung G. W. Rensvold R. B. (2000). Assessing extreme and acquiescence response sets in cross-cultural research using structural equations modeling. *J. Cross Cult. Psychol.* 31 187–212. 10.1177/0022022100031002003

[B17] Church A. T. Alvarez J. M. Mai N. T. French B. F. Katigbak M. S. Ortiz F. A. (2011). Are cross-cultural comparisons of personality profiles meaningful? Differential item and facet functioning in the Revised NEO Personality Inventory. *J. Pers. Soc. Psychol.* 101 1068–1089. 10.1037/a0025290 21910552

[B18] Church A. T. Lonner W. J. (1998). The cross-cultural perspective in the study of personality rationale and current research. *J. Cross Cult. Psychol.* 29 32–62. 10.1177/0022022198291003

[B19] Costa P. Jr. Terracciano A. McCrae R. R. (2001). Gender differences in personality traits across cultures: robust and surprising findings. *J. Pers. Soc. Psychol.* 81 322–331. 10.1037/0022-3514.81.2.322 11519935

[B20] Costa P. T. McCrae R. R. (1992). *Revised NEO Personality Inventory (NEO PI-R) and NEO Five-Factor Inventory (NEO FFI): Professional Manual.* (Odessa, FL: Psychological Assessment Resources, Inc.), 102

[B21] Dash S. Bruning E. Acharya M. (2009). The effect of power distance and individualism on service quality expectations in banking: a two-country individual-and national-cultural comparison. *Int. J. Bank Mark.* 27 336–358. 10.1108/02652320910979870

[B22] Davidson A. R. Jaccard J. J. Triandis H. C. Morales M. L. Diaz-Guerrero R. (1976). Cross-cultural model testing: toward a solution of the etic-emic dilemma. *Int. J. Psychol.* 11 1–13. 10.1080/00207597608247343

[B23] Davis M. H. (1983). Measuring individual differences in empathy: evidence for a multidimensional approach. *J. Pers. Soc. Psychol.* 44 113–126. 10.1037/0022-3514.44.1.113

[B24] Deary I. J. Matthews G. (1993). Traits are alive and well. *Psychologist* 6 299–311.

[B25] De Raad B. (1998). Five big, big five issues: rationale, content, structure, status, and crosscultural assessment. *Eur. Psychol.* 3 113–124. 10.1027/1016-9040.3.2.113

[B26] De Raad B. Perugini M. Hrebícková M. Szarota P. (1998). Lingua franca of personality taxonomies and structures based on the psycholexical approach. *J. Cross Cult. Psychol.* 29 212–232. 10.1177/0022022198291011

[B27] de Waal F. B. (2008). Putting the altruism back into altruism: the evolution of empathy. *Annu. Rev. Psychol.* 59 279–300. 10.1146/annurev.psych.59.103006.093625 17550343

[B28] del Barrio V. Aluja A. García L. F. (2004b). Bryant’s empathy index for children and adolescents: psychometric properties in the spanish language. *Psychol. Rep.* 95 257–262. 10.2466/PR0.95.5.257-262 15460381

[B29] del Barrio V. D. Aluja A. García L. F. (2004a). Relationship between empathy and the Big Five Personality traits in a sample of Spanish adolescents. *Soc. Behav. Pers.* 32 677–681. 10.2224/sbp.2004.32.7.677

[B30] Derntl B. Finkelmeyer A. Eickhoff S. Kellermann T. Falkenberg D. I. Schneider F. (2010). Multidimensional assessment of empathic abilities: neural correlates and gender differences. *Psychoneuroendocrinology* 35 67–82. 10.1016/j.psyneuen.2009.10.006 19914001

[B31] Duan C. Wei M. Wang L. (2008). The role of individualism-collectivism in empathy: an exploratory study. *Asian J. Counsel.* 15 57–81.

[B32] Dyche L. Zayas L. H. (2001). Cross-cultural empathy and training the contemporary psychotherapist. *Clin. Soc. Work J.* 29 245–258. 10.1023/A:1010407728614

[B33] Enzmann D. (1996). *Gestreßt, Erschöpft Oder Ausgebrannt? Einflüsse von Arbeitssituationen, Empathie und Coping auf den Burnoutprozeß.* München: Profil Publisher.

[B34] Faul F. Erdfelder E. Buchner A. Lang A.-G. (2009). Statistical power analyses using G*Power 3.1: tests for correlation and regression analyses. *Behav. Res. Methods* 41 1149–1160. 10.3758/BRM.41.4.1149 19897823

[B35] Ford M. E. (1982). Social cognition and social competence in adolescence. *Dev. Psychol.* 18 323–340. 10.1037/0012-1649.18.3.323

[B36] Goldberg L. R. (1999). “A broad-bandwidth, public domain Personality inventory measuring the lower-level facets of several five-factor models,” in *Personality Psychology in Europe* Vol. 7 eds Mervielde I. Deary I. De Fruyt F. Ostendorf F. (Tilburg: Tilburg University Press), 7–28.

[B37] Graziano W. G. Eisenberg N. (1997). “Agreeableness: a dimension of personality,” in *Handbook of Personality Psychology*, eds Hogan R. Johnson J. Briggs S. R. (San Diego, CA: Academic Press), 795–824.

[B38] Graziano W. G. Habashi M. M. Sheese B. E. Tobin R. M. (2007). Agreeableness, empathy, and helping: a person × situation perspective. *J. Pers. Soc. Psychol.* 93 583–599. 10.1037/0022-3514.93.4.583 17892333

[B39] Heine S. J. Lehman D. R. Peng K. Greenholtz J. (2002). What’s wrong with cross-cultural comparisons of subjective Likert scales? The reference-group effect. *J. Pers. Soc. Psychol.* 82 903–918. 10.1037/0022-3514.82.6.903 12051579

[B40] Hofstede G. (1979). “Value systems in forty countries: interpretation, validation, and consequences for theory,” in *Cross-Cultural Contributions to Psychology*, eds Eckensberger L. H. Lonner W. J. Poortinga Y. H. (Lisse: Swets and Zeitlinger).

[B41] Hofstede G. (1980). *Culture’s Consequences: International Differences in Work-Related Values.* Beverly Hills, CA: Sage Publication.

[B42] Hofstede G. (1982). “Dimensions of national cultures,” in *Diversity and Unity in Cross-Cultural Psychology*, eds Rath R. Asthana H. S. Sinha D. Sinha J. B. H. (Lisse: Swets and Zeitlinger).

[B43] Hofstede G. (1983). “Dimensions of national cultures in fifty countries and three regions,” in *Expiscations in Cross-Cultural Psychology*, eds Deregowksi J. B. Dziurawiec S. Annis R. C. (Lisse: Swets and Zeitlinger).

[B44] Hofstede G. (2001). *Culture’s Consequences: Comparing Values, Behaviors, Institutions, and Organizations Across Nations*, 2nd. Edn Thousand Oaks, CA: Sage Publication.

[B45] Hofstede G. Bond M. H. (1984). Hofstede’s culture dimensions an independent validation using rokeach’s value survey. *J. Cross Cult. Psychol.* 15 417–433. 10.1177/0022002184015004003

[B46] John O. P. Srivastava S. (1999). “The big five trait taxonomy: history, measurement, and theoretical perspectives,” in *Handbook of Personality: Theory and Research*, Vol. 2 eds Lawrence A. P. Oliver P. J. (Berkeley, CA: University of California at Berkeley), 102–138.

[B47] Jolijn Hendriks A. A. Perugini M. Angleitner A. Ostendorf F. Johnson J. A. De Fruyt F. (2003). The five-factor personality inventory: cross-cultural generalizability across 13 countries. *Eur. J. Pers.* 17 347–373. 10.1002/per.491

[B48] Juni S. (1996). “Review of the revised NEO personality inventory,” in *12th Mental Measurement Yearbook*, eds Conoley J. C. Impara J. C. (Lincoln, NB: University of Nebraska Press), 863–868.

[B49] Knafo A. Zahn-Waxler C. van Hulle C. Robinson J. L. Rhee S. H. (2008). The developmental origins of a disposition toward empathy: genetic and environmental contributions. *Emotion* 8 737–752. 10.1037/a0014179 19102585

[B50] Lawrence E. J. Shaw P. Baker D. Baron-Cohen S. David A. S. (2004). Measuring empathy: reliability and validity of the Empathy Quotient. *Psychol. Med.* 34 911–919. 10.1017/S0033291703001624 15500311

[B51] Lennon R. Eisenberg N. (1987). “Gender and age differences in empathy and sympathy,” in *Empathy and Its Development*, eds Eisenberg N. Strayer J. (Cambridge, CA: University Press), 195–217.

[B52] MacLean P. D. (1985). Brain evolution relating to family, play, and the separation call. *Arch. Gen. Psychiatry* 42 405–417. 10.1001/archpsyc.1985.01790270095011 3977559

[B53] McCrae R. R. Allik J. (2002). *The Five-Factor Model of Personality Across Cultures.* New York, NY: Kluwer Academic.

[B54] McCrae R. R. Costa P. T. (1985). Comparison of EPI and psychoticism scales with measures of the five-factor model of personality. *Pers. Individ. Differ.* 6 587–597. 10.1016/0191-8869(85)90008-X

[B55] McCrae R. R. Costa P. T. (2003). *Personality in Adulthood: A Five-Factor Theory Perspective.* New York City, NY: Guilford Press.

[B56] McCrae R. R. Costa P. T. Jr. Yik M. S. M. (1996). “Universal aspects of Chinese personality structure,” in *The Handbook of Chinese Psychology* ed Bond M. H. (Hong Kong: Oxford University Press), 189–207.

[B57] Melchers M. Montag C. Markett S. Reuter M. (2015). Assessment of empathy via self-report and behavioral paradigms: data on convergent and discriminant validity. *Cogn. Neuropsychiatry* 20 157–171. 10.1080/13546805.2014.991781 25530230

[B58] Moore B. S. (1990). The origins and development of empathy. *Motiv. Emot.* 14 75–80. 10.1007/BF00991636

[B59] Muncer S. J. Ling J. (2006). Psychometric analysis of the empathy quotient (EQ) scale. *Pers. Individ. Differ.* 40 1111–1119. 10.1016/j.paid.2005.09.020

[B60] Nettle D. (2007). Empathizing and systemizing: what are they, and what do they contribute to our understanding of psychological sex differences? *Br. J. Psychol.* 98 237–255. 10.1348/000712606X117612 17456271

[B61] Nye C. D. Roberts B. W. Saucier G. Zhou X. (2008). Testing the measurement equivalence of personality adjective items across cultures. *J. Res. Pers.* 42 1524–1536. 10.1016/j.jrp.2008.07.004

[B62] Pérez-Albéniz A. De Paúl J. Etxeberría J. Montes M. P. Torres E. (2003). Adaptación de interpersonal reactivity index (IRI) al español. *Psicothema* 15 267–272.

[B63] Poortinga Y. H. Van Hemert D. A. (2001). Personality and culture: demarcating between the common and the unique. *J. Pers.* 69 1033–1060. 10.1111/1467-6494.696174 11767817

[B64] Realo A. Allik J. Vadi M. (1997). The hierarchical structure of collectivism. *J. Res. Pers.* 31 93–116. 10.1006/jrpe.1997.2170

[B65] Realo A. Luik M. (2002). On the relationship between collectivism and empathy in the context of personality traits. *Trames J. Humanit. Soc.* 6 218–233.

[B66] Richendoller N. R. Weaver J. B. (1994). Exploring the links between personality and empathic response style. *Pers. Individ. Differ.* 17 303–311. 10.1016/0191-8869(94)90278-X

[B67] Roberts B. W. Walton K. E. Viechtbauer W. (2006). Patterns of mean-level change in personality traits across the life course: a meta-analysis of longitudinal studies. *Psychol. Bull.* 132 1–25. 10.1037/0033-2909.132.1.1 16435954

[B68] Rosnow R. L. Rosenthal R. (2003). Effect sizes for experimenting psychologists. *Can. J. Exp. Psychol.* 57 221–237. 10.1037/h0087427 14596479

[B69] Schmitt D. P. Allik J. McCrae R. R. Benet-Martínez V. (2007). The geographic distribution of Big Five Personality traits patterns and profiles of human self-description across 56 nations. *J. Cross-Cult. Psychol.* 38 173–212. 10.1177/0022022106297299

[B70] Schmitt D. P. Realo A. Voracek M. Allik J. (2008). Why can’t a man be more like a woman? Sex differences in Big Five personality traits across 55 cultures. *J. Pers. Soc. Psychol.* 94 168–182. 10.1037/0022-3514.94.1.168 18179326

[B71] Shaver P. R. Brennan K. A. (1992). Attachment styles and the “Big Five” Personality traits: their connections with each other and with romantic relationship outcomes. *Pers. Soc. Psychol. Bull.* 18 536–545. 10.1177/0146167292185003

[B72] Siu A. M. Shek D. T. (2005). Validation of the interpersonal reactivity index in a Chinese context. *Res. Soc. Work Pract.* 15 118–126. 10.1177/1049731504270384

[B73] Triandis H. C. (1989). The self and social behavior in differing cultural contexts. *Psychol. Rev.* 96 506–520. 10.1037/0033-295X.96.3.506

[B74] Triandis H. C. (1995). *Individualism and Collectivism.* Boulder, CO: Westview.

[B75] Trommsdorff G. (1995). “Person-context relations as developmental conditions for empathy and prosocial action: a cross-cultural analysis,” in *Development of Person-Context Relations*, eds Kindermann T. A. Valsiner J. (Hillsdale, NJ: Erlbaum), 113–146.

[B76] van de Vijver F. J. R. (2000). “The nature of bias,” in *Handbook of Cross-Cultural and Multicultural Personality Assessment*, ed Dana R. H. (Mahwah, NJ: Lawrence Erlbaum), 87–106.

[B77] Van de Vijver F. Leung K. (2001). Personality in cultural context: methodological issues. *J. Pers.* 69 1007–1031. 10.1111/1467-6494.696173 11767816

[B78] Van der Zee K. Thijs M. Schakel L. (2002). The relationship of emotional intelligence with academic intelligence and the Big Five. *Eur. J. Pers.* 16 103–125. 10.1002/per.434

[B79] Wakabayashi A. Kawashima H. (2015). Is empathizing in the E–S theory similar to agreeableness? The relationship between the EQ and SQ and major Personality domains. *Pers. Individ. Differ.* 76 88–93. 10.1016/j.paid.2014.11.030

[B80] Walter H. (2012). Social cognitive neuroscience of empathy: concepts, circuits, and genes. *Emot. Rev.* 4 9–17. 10.1177/1754073911421379

[B81] Ward C. Leong C.-H. Low M. (2004). Personality and sojourner adjustment an exploration of the big five and the cultural fit proposition. *J. Cross Cult. Psychol.* 35 137–151. 10.1177/0022022103260719

[B82] Whiteside S. P. Lynam D. R. (2001). The five factor model and impulsivity: using a structural model of Personality to understand impulsivity. *Pers. Individ. Differ.* 30 669–689. 10.1016/S0191-8869(00)00064-7

